# Case Series of Abdominal Actinomycosis: An Old Diagnostic Conundrum

**DOI:** 10.7759/cureus.69763

**Published:** 2024-09-19

**Authors:** Firas Ayoub, Amani Asour, Anur Miah

**Affiliations:** 1 General and Colorectal Surgery, Medway Maritime Hospital, Gillingham, GBR; 2 General and Colorectal Surgery, Dartford and Gravesham NHS Trust, Kent, GBR; 3 Medical Education, Queen Mary University of London, London, GBR; 4 General Surgery, Dartford and Gravesham NHS Trust, Kent, GBR; 5 General and Colorectal Surgery, Lewisham and Greenwich NHS Trust, London, GBR

**Keywords:** abdominal pain, actinomycosis, challenging diagnosis, colorectal, hemicolectomy, infectious disease, intrabdominal infection, surgery

## Abstract

Abdominal actinomycosis is a rare disease caused by the *Actinomyces* bacteria. We report a case series of two similar cases with a mismatch between the initial differential diagnosis, the radiological findings, the surgical findings, and the actual histological result. The first case is a 25-year-old woman with a month’s history of right-sided abdominal pain. The diagnostic workup including computed tomography (CT) showed possible acute appendicitis, in addition to ascending colon looking abnormal with inflammatory changes and adjacent peritoneal nodules. A diagnostic laparoscopy revealed a normal-looking appendix and a hard mass involving the ascending colon potentially of inflammatory or malignant origin. A laparoscopic right hemicolectomy was performed. Surprisingly, the histology concluded actinomycosis. The second case is a 38-year-old woman with a six-week history of lower abdominal pain and right iliac fossa (RIF) swelling. The diagnostic workup including a CT scan showed a complex mass suggesting complicated appendicitis, pelvic inflammatory disease, or neoplastic process. Furthermore, magnetic resonance imaging (MRI) of the pelvis showed complex bilateral cystic lesions in both iliac fossas with an abscess formation within the subcutaneous fat overlying the lower pelvis. The patient underwent an ultrasound (US)-guided drainage of the subcutaneous pelvic collection and a diagnostic laparoscopy that showed an inflamed mass with dense adhesions to the anterior abdominal wall. Intraoperative biopsies were taken to confirm the diagnosis, which ultimately confirmed severe pelvic actinomycosis. Both patients were discharged home and received extended antibiotic treatment according to microbiology guidelines with good outcomes. Both cases are interesting because there was a mismatch between the initial differential diagnosis, the radiological findings, the intraoperative findings, and the final histology. Reported cases in the literature highlight the diagnostic challenges of abdominal actinomycosis, as it often presents with atypical symptoms and imaging findings sometimes mimic malignancy leading to surgical intervention.

## Introduction

Actinomycosis is a relatively rare bacterial disease that primarily involves the cervicofacial areas in susceptible populations with rare abdominal involvement [1]. The most common abdominal sites are the appendix, caecum, and colon [2]. Actinomycosis has been described as the most misdiagnosed disease because no other disease is so often missed by experienced physicians [3]. Once actinomycosis involves the abdomen, the diagnosis is usually challenging and often mimics other differentials such as acute appendicitis, inflammatory bowel disease, and malignancy [4]. We report two similar cases with a mismatch between the initial differential diagnosis, the radiological findings, the surgical findings, and the actual histological result.

## Case presentation

Case 1

A 25-year-old female was referred by her general practitioner to the acute surgical team with a four-week history of right-sided abdominal pain. The pain originally started near the umbilical area and gradually worsened and radiated to the right iliac fossa. The patient described the pain as a sharp severe stabbing constant pain at the right side of her abdomen. She had no symptoms indicating genitourinary infection or any other source of sepsis. The patient had a history of an intrauterine device (IUD) for two years that was removed eight months prior to her presentation. She was previously fit and healthy and was not on regular medications other than simple analgesia taken when required to control her pain. She smoked 10 cigarettes a day for the last five years and drank alcohol occasionally. Her body mass index was within the normal range.

On physical examination, the patient looked unwell and was noted to have tachycardia of 106 bpm and pyrexia of 38.3 °C. Her abdomen was soft and tender at the right lower quadrant with minimal guarding but without any signs of peritonitis. A digital rectal examination (DRE) performed was normal. The patient had a normal chest examination. On admission, the patient had her blood taken for routine analysis, cultures, and a transvaginal ultrasound (TVUS).

**Figure 1:**
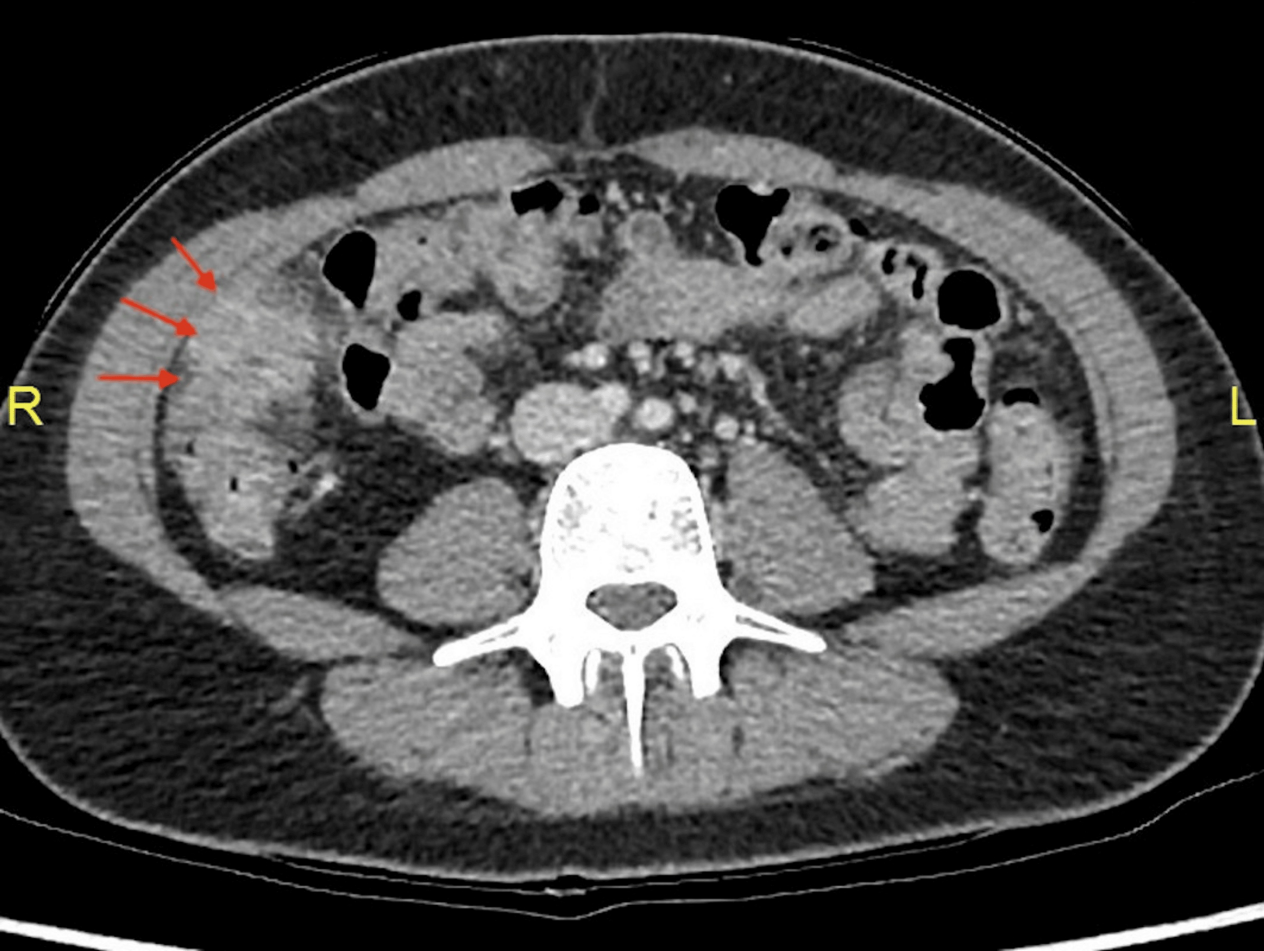
Axial image of the CT abdomen showing an infiltrative inflammatory mass adjacent to the ascending colon.

Initial laboratory results showed a C-reactive protein (CRP) of 204 mg/L and white cell count (WCC) of 15.3/L with neutrophilia of 12/L (Table 1). The TVUS did not reveal any gross abnormality. On account of the clinical history, physical exam, and laboratory findings, the patient was sent for computed tomography of the abdomen and pelvis (CTAP) with contrast to identify any intra-abdominal sepsis. Since the patient presented during the coronavirus disease 2019 (COVID-19) outbreak, the CT scan included the chest to rule out any lung disease related to coronavirus. Interestingly, the CT showed features suggesting acute appendicitis, and multiple peritoneal lesions were seen in association with an ascending colon mass, the nature of which was uncertain (Figure 1). The patient was notified of the CT result, and on account of her clinical presentation, the CT findings, and the unclear nature of the underlying pathology, she was listed for a diagnostic laparoscopy on the emergency surgical list.

**Table 1:** Laboratory values of case 1

Test	Result	Units	Normal Range
White cell count (WCC)	15.3	x10*9/L	(4.0-11.0)
Neutrophils	12	x10*9/L	(2.0-7.0)
C-reactive protein (CRP)	204	mg/L	(0.0-5.0)

The most likely differential diagnosis was acute appendicitis since it is common in this age group. Taking into account the patient's history and examination, a gynecological cause was felt unlikely, but could not be absolutely ruled out. Inflammatory bowel disease was also possible as the CT demonstrated thickening of the ascending colon and the chronicity of symptoms. Lastly, malignancy was also considered in light of the history and peritoneal abnormality on CT and intraoperative findings. Therefore, a diagnostic laparoscopy was inevitable.

**Figure 2:**
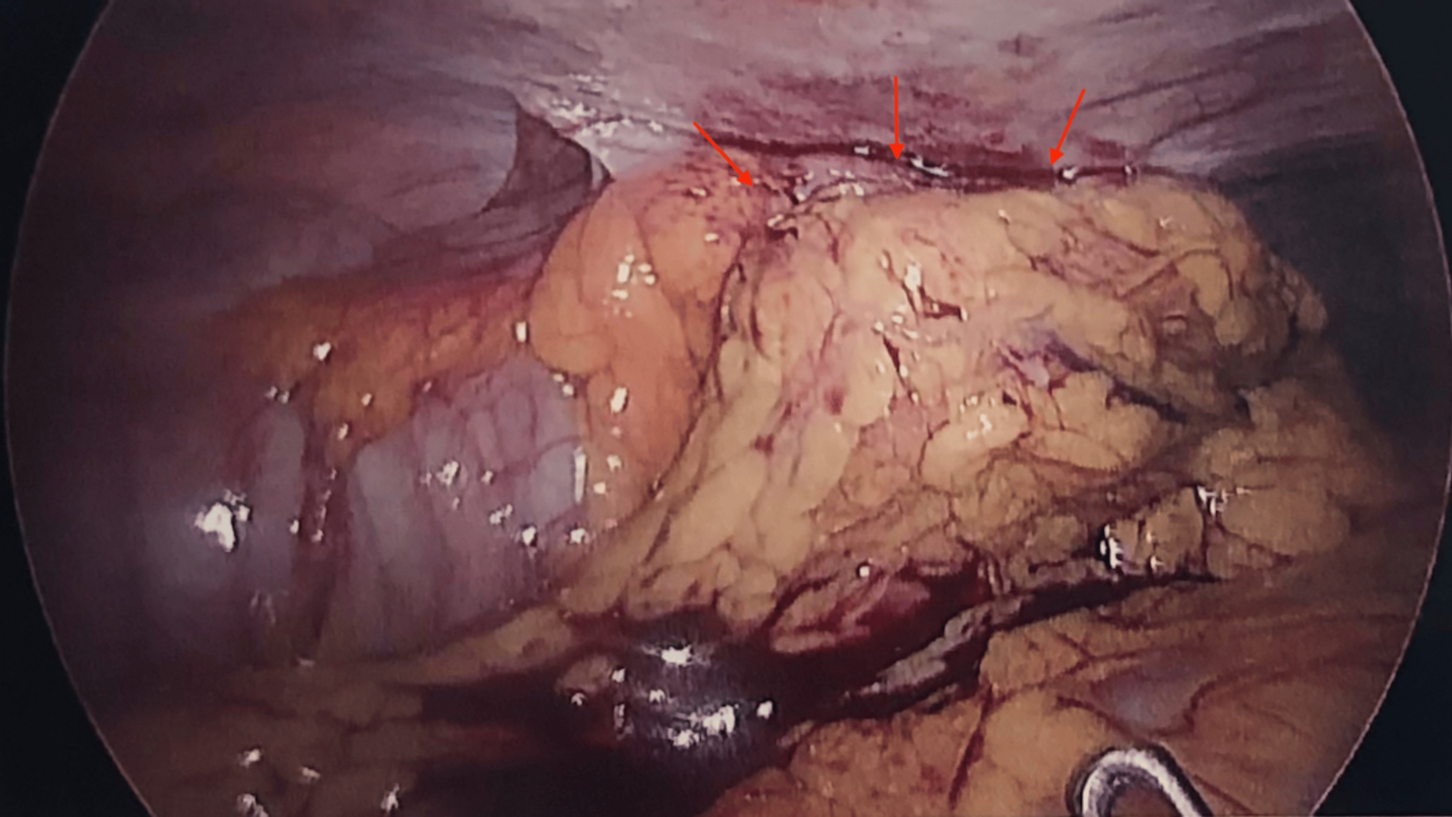
Intraoperative image showing an ascending colon mass.

The patient was managed preoperatively with intravenous fluids and broad-spectrum antibiotics including co-amoxiclav and gentamicin. The CT findings were thoroughly explained to the patient, as well as the plan for a diagnostic laparoscopy and surgical intervention. During the diagnostic laparoscopy, a normal-looking appendix was easily identified. However, there was a hard mass involving the ascending colon stuck onto the anterior abdominal wall wrapped in omental adhesions potentially of inflammatory or malignant origin (Figure 2). A laparoscopic right hemicolectomy was performed with an en-block disc of the peritoneum and omental resection with primary anastomosis, and the specimen was sent for urgent histology.

**Figure 3:**
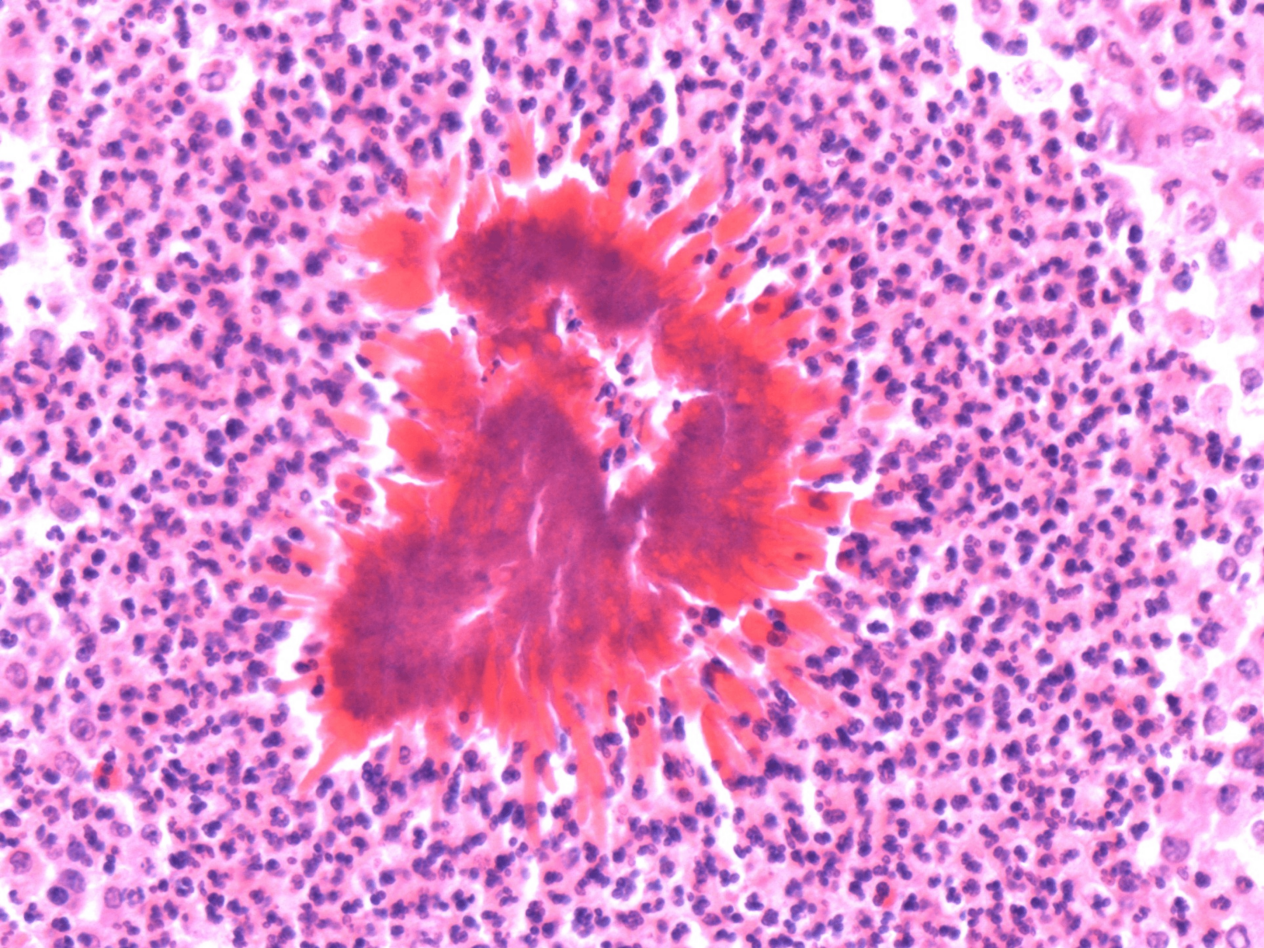
Histopathology confirming Sulfur granules.

The patient had an uneventful postoperative recovery and was discharged on day six of her admission with a plan for a follow-up in the surgical outpatient clinic. On histology, "sulfur" granules were noticed; consisting of an irregularly rounded cluster of bacteria (filaments) bordered by eosinophilic, cube, and rectangular-like projections called a Splendore-Hoeppli material (Figure 3). Histology confirmed the diagnosis of abdominal actinomycosis. The patient completed a four-week course of oral antibiotics (amoxicillin 500 mg three times per day) as per the microbiologist's recommendation. She was later reviewed in the outpatient clinic and reported a good clinical response.

Case 2

A 38-year-old female was brought by the ambulance to the emergency department complaining of a six-week history of worsening lower abdominal pain and a swelling in the right iliac fossa (RIF). The pain was associated with a prolonged period of constipation, nausea, and malaise that affected her quality of life. She had no genitourinary symptoms. Of note, the patient had a history of IUD that was removed two months prior to the onset of her symptoms. She also had a background history of migraines and penicillin allergy; her regular medications included sumatriptan, propranolol, and topiramate. She smoked 10 cigarettes a day and drank alcohol occasionally. Her BMI was within normal range.

Three weeks prior to this presentation, the patient attended the emergency department for a similar pain; at that time, a CT of her kidneys-ureters-bladder (KUB) showed a suspicious nodule at the right anterior bladder wall. This had been investigated with an urgent cystoscopy and a CT urogram that ruled out bladder disease. At the time, she was reassured and discharged home with analgesia.

On physical examination, the patient was in agony and was noted to have a low blood pressure (96/55 mmHg) with the rest of the vitals being within normal range. Her abdomen was soft but tender at the right lower quadrant with a palpable mass in the right iliac fossa and no peritonism. A digital rectal examination (DRE) performed was unremarkable.

**Figure 4:**
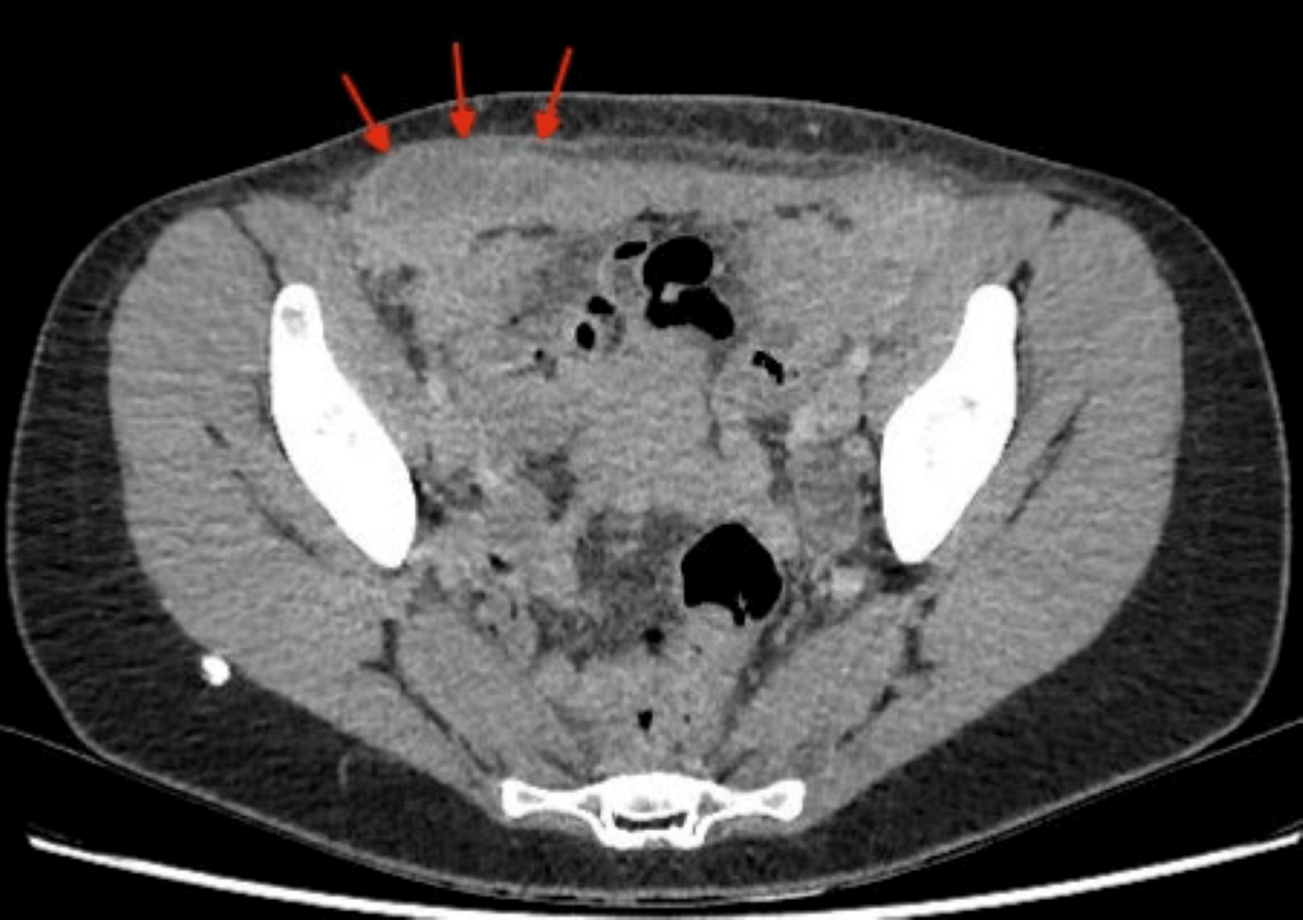
Axial image of CT abdomen showing a nonspecific inflammation in the peritoneal cavity.

Initial laboratory results showed a CRP of 177 mg/L and a WCC of 15.9/L with neutrophilia of 12.6/L (Table 2). On account of the clinical history, physical examination, and laboratory findings, the patient underwent a series of investigations including multiple imaging tests during her admission to the surgical ward. Initially, a CTAP with contrast showed features of an evolving abscess within the right abdominal wall muscles and a nonspecific inflammation in the peritoneal cavity in the RIF adjacent to the caecum extending to the right ovary (Figure 4). It also showed inflammation in the left lower anterior abdominal wall muscles.

**Table 2:** Laboratory values of case 2

Test	Result	Units	Normal Range
white cell count (WCC)	15.9	x10*9/L	(4.0-11.0)
Neutrophils	12.6	x10*9/L	(2.0-7.0)
C-reactive protein (CRP)	177	mg/L	(0.0-5.0)

**Figure 5:**
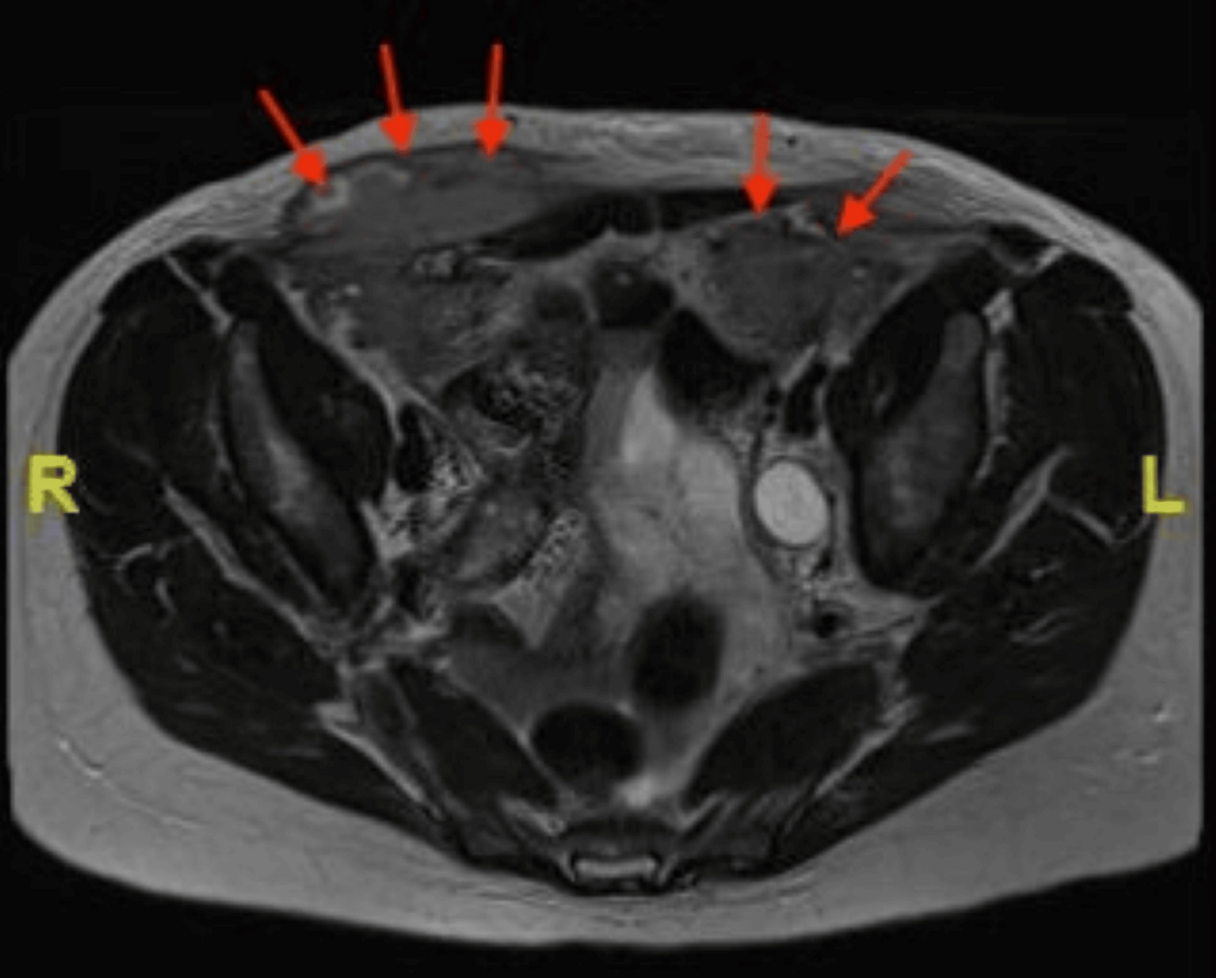
Axial MRI pelvis showing solid lesions within both iliac fossas.

The CT was then compared to the patient’s recent CT KUB and showed that the nodule next to the bladder had increased in size. Due to the indeterminate diagnostic findings of the CTAP, the patient underwent a magnetic resonance imaging (MRI) of the pelvis that demonstrated cystic solid lesions within both iliac fossas and subcutaneous components consistent with abscess formation and collections in the fat overlying the low pelvic area (Figure 5). Subsequently, a transvaginal ultrasound (TVUS) took place and ruled out adnexal pathology. The investigations also included negative blood cultures and a raised fecal calprotectin of 250 µg/g. 

Given the findings, the patient was scheduled for an US-guided drainage of the anterior abdominal wall collection. Pus (500 ml) was drained, and cultures did not grow any microorganisms. The patient was discussed in the multidisciplinary team meeting, and it was decided to undergo diagnostic laparoscopy and tissue biopsy.

The patient had an ongoing intra-abdominal inflammation that could be a result of various pathologies. However, the diagnosis was not straightforward. As the CTAP showed a nonspecific inflammation extending to the right ovary, the initial clinical impression was a gynaecologic pathology. Further investigations were obtained including MRI pelvis and TVUS. In addition, the patient was reviewed by the gynecology team who deemed a gynecologic cause for this presentation unlikely.

Given the fact that the imaging was inconclusive and showed features that could possibly indicate malignant pathology, an urgent multidisciplinary team meeting was arranged. The outcome of the meeting was an urgent diagnostic laparoscopy, washout of the abdominal cavity, and tissue biopsy.

The patient was managed preoperatively with intravenous fluids and broad-spectrum antibiotics including ciprofloxacin and metronidazole as she was penicillin allergic. The imaging findings were thoroughly explained to the patient, as well as the plan for diagnostic laparoscopy and surgical intervention. The patient underwent an ultrasound-guided drainage of the anterior abdominal wall collection.

The diagnostic laparoscopy revealed bilateral pelvic inflammatory masses possibly involving the bowel with dense adhesions to the anterior abdominal wall. There was also blood-stained fluid in the pelvis mainly in the pouch of Douglas. Only the left-sided adhesions were bluntly dissected as the right-sided adhesions seemed to involve the bowel. The intrabdominal cavity and pelvis were thoroughly washed out with normal saline 0.9%, and a drain was inserted into the left pelvic area. The patient had an uneventful postoperative recovery and was discharged home on oral antibiotics (clarithromycin 500 mg twice daily).

**Figure 6:**
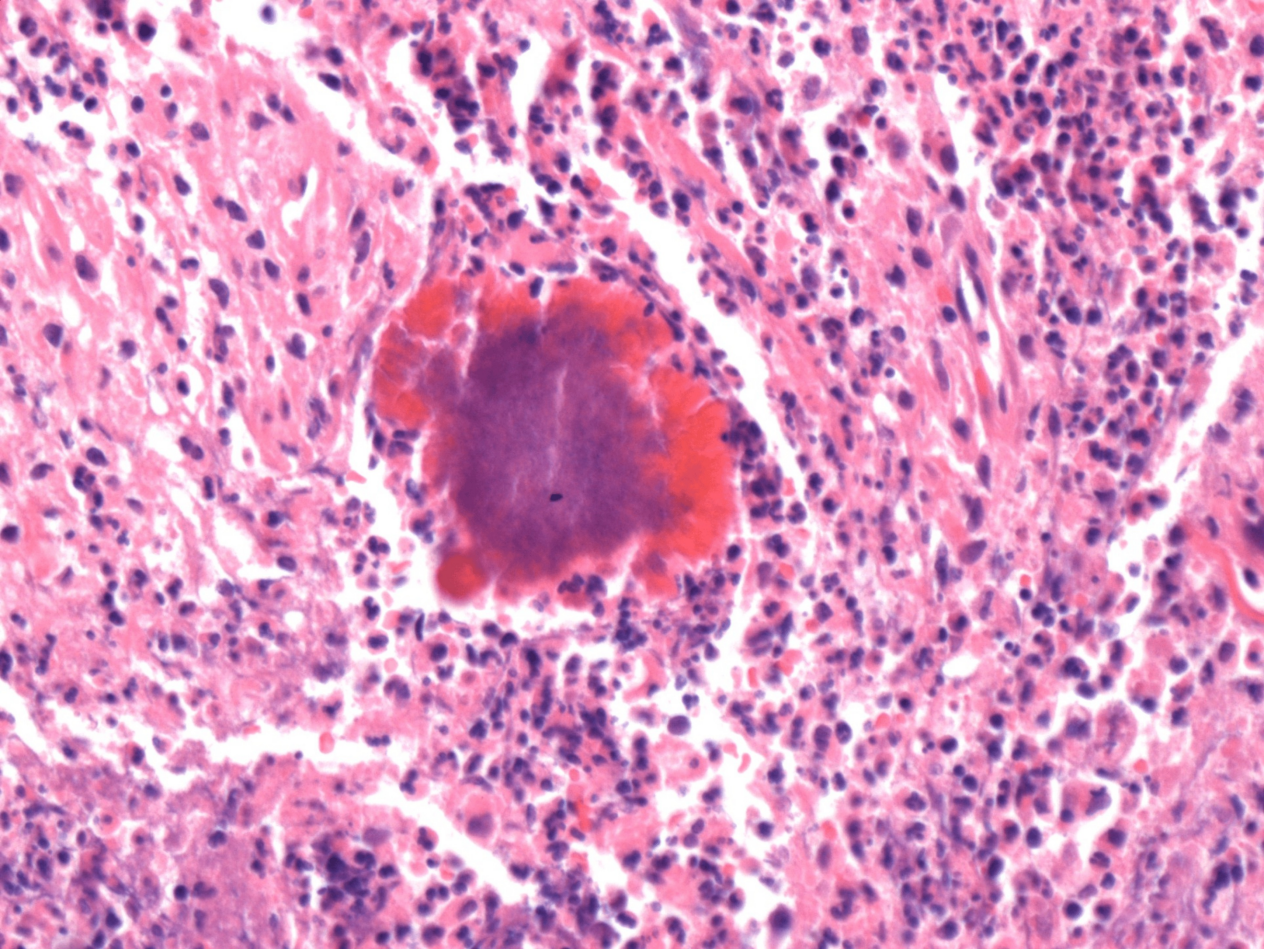
Histopathological finding of actinomycosis.

The patient stayed in the hospital for 17 days. The tissue biopsies showed severe active and chronic actinomycosis (Figure 6). The patient was then reviewed by the infectious diseases team who suggested a six-month course of oral clarithromycin. The patient had regular follow-ups with both the surgical and infectious diseases team and close monitoring of her inflammatory markers. Two months after her discharge from the hospital, the patient showed a significant clinical response to the antibiotic treatment.

## Discussion

Actinomycosis has been reported in the literature for over a century and was first described in 1879 [4]. It is a rare and chronic infectious granulomatous disease caused by filamentous anaerobic gram-positive *Actinomyces* bacteria [5,6]. *Actinomyces* species are usually found in the human commensal flora of the oropharynx, gastrointestinal tract, and urogenital system [7]. Histologically, this is characterized by suppurative and granulomatous inflammation in addition to multiple abscesses and sinus tract formation that may discharge sulfur granules [6]. In 1970, the reported annual incidence of actinomycosis in Cleveland USA was 1:300,000, but the incidence has declined with the improvement of dental hygiene and widespread usage of antibiotics [8]. *Actinomyces* infections are more common in men than women with a ratio of 3:1. The disease can affect people of all ages (mostly 20-50 years of age) and has no racial predilection, and once identified, its prognosis is excellent with appropriate antibiotic treatment [6].

Abdominal and pelvic actinomycosis accounts for 10-20% of reported cases, and it is the third most common site following cervicofacial (50-65%) and thoracic (15-30%) [4,6]. The appendix, caecum, and colon are the most common sites where abdominal actinomycosis can present. Once the infection occurs, it can take weeks to years for the disease to evolve after mucosal disruption [2,7]. Recent abdominal surgery (i.e., appendicectomy, perforated colonic diverticulitis) or ingestion of foreign body (i.e., fish bone), breaches the colonic mucosa, which could lead to *Actinomyces* spread and invasion of adjacent tissues [6,7]. In general, abdominal actinomycosis is rare and often presents with nonspecific symptoms making the diagnosis of actinomycosis difficult. The disease is frequently misdiagnosed because the insidious bacterial infection has a tendency to involve surrounding tissues and mimic malignancy [2,7,9].

In 2011, Sung et al. published a study about the clinical features of abdominal actinomycosis and its therapeutic outcomes in 23 cases between 1994 and 2010. All of the cases in the study had a surgical operation (50% emergency surgery for peritonitis) and a common finding identified in the preoperative stage was a malignant mass seen on CT. None of the cases had a percutaneous biopsy. In all cases, the diagnostic standard was histological confirmation after surgery [9]. Ketata et al. in 2010 reviewed seven cases of abdominal actinomycosis that were treated surgically. In his review, the diagnosis of abdominal actinomycosis was only confirmed on histology [10].

In 2003, Walgender et al. conducted a literature review over a decade and concluded that preoperative clinical and radiological diagnosis is rare and the laboratory tests and symptoms are nonspecific [11]. On the other hand, Ha et al. studied the CT images of 10 cases with histologically proven actinomycosis. The main common feature described in these CT findings was the infiltrative nature of the disease and contrast enhancement of the wall or components of the infiltrative masses. The study concluded that despite the CT findings being nonspecific, a suspicion of actinomycosis should be raised [12]. However, a patient presenting with abdominal sepsis with peritoneal irritation will usually undergo surgical intervention when the diagnosis is unclear, even if it is a diagnostic laparoscopy in the first instance.

In conclusion, abdominal actinomycosis is a rare and often chronic infectious disease caused by gram-positive *Actinomyces* bacteria. The rarity and unusual presentation of the disease make it difficult to include actinomycosis in the initial differential diagnosis when treating patients with abdominal sepsis. The actual diagnosis is usually reached in these cases when histology is reported from the surgical specimen. Once the diagnosis is confirmed, a long course of antibiotics is required to eradicate the disease.

## Conclusions

Abdominal actinomycosis is a relatively rare bacterial infection that can present atypically and can mimic other surgical conditions including malignancy making diagnosis challenging. Actinomycosis ought to be at least borne in mind as a potential differential diagnosis in unusual presentations of abdominal sepsis and atypical radiological findings. Surgical intervention in the acute setting is often inevitable.
